# Diaspora as partners: strengthening resilience of health systems and communities amidst aid volatility

**DOI:** 10.1136/bmjgh-2025-019622

**Published:** 2025-06-19

**Authors:** Alaa Dafallah, Sophie Witter, ReBUILD for Resilience Consortium

**Affiliations:** 1 Centre for Global Health Research, Nuffield Department of Medicine, University of Oxford, Oxford, UK; 2 ReBUILD Consortium, Liverpool, UK; 3 Institute for Global Health and Development, Queen Margaret University Edinburgh, Edinburgh, UK

**Keywords:** Health policy

**Summary Box**

- Global aid volatility threatens health systems and communities in low- and middle-income countries (LMICs) and aid-dependent fragile settings, disrupting critical essential services and programmes.
- Diaspora financial, human and social capital represent critical resilience capabilities for communities and health systems in LMICs and aid-dependent fragile settings.
- Harnessing diaspora capabilities for resilience of health systems and communities requires recognition, integration and evidence-based action.
- The shifting aid landscape necessitates a reimagining of health financing and global health partnerships, where diaspora are key partners.

## Introduction

 The global aid landscape is experiencing unprecedented volatility. Aid has been cut, abruptly, with devastating consequences for health systems and communities across low- and middle-income countries (LMICs), particularly aid-dependent fragile settings. The US government’s January 2025 stop work order froze $40 billion in foreign assistance, disrupting 1400+ programmes across 133 countries.^1^ Recently, the UK government announced reducing aid budgets from 0.5% to 0.33% of gross national income (GNI), effectively halving their overseas development assistance (ODA) commitments.^2^ The Netherlands and Sweden had previously announced significant aid cuts, and it is likely that more countries will follow suit.

These disruptions have spurred critical conversations on domestic resource mobilisation and sustainable financing for essential health services and health systems in LMICs.^3^ We bring to this conversation an urgent consideration: the critical, overlooked and underutilised value of diaspora and their contributions for health systems in LMICs and fragile and shock-prone settings. We discuss modalities through which diaspora contributes to the resilience of health systems and communities in these contexts, concluding with recommendations to strengthen the role of diaspora in this space.

## Diaspora remittances: invisible aid

Diaspora contributions to communities and health systems in countries of origin are historical, substantial and multifaceted. Remittances stand as the largest source of external financing for LMICs since 2015, with flows reaching an estimated $669 billion in 2023, exceeding the combined value of overseas development assistance and development finance.^4^ Africa alone received over $90 billion in remittances in 2023, though actual figures are likely higher due to prevalent informal transfer channels.^5^ In fragile settings, remittances constitute a significant fraction of national gross domestic product (GDP), e.g. 23.5% in Somalia and 31% in Lebanon, with a substantial portion supporting household expenditures and local health facilities during the ongoing humanitarian crises.^6^

Beyond their sheer magnitude, remittances have distinct advantages over traditional aid.^7^ They demonstrate relatively greater countercyclical stability, as evidenced during COVID-19, where contrary to predictions, remittances either remained stable or increased.^8^ They achieve higher absorption rates (80–90% compared with 50% for aid) due to reduced overhead costs.^7^ Furthermore, remittances form agile, responsive, needs-based informal social protection systems, leveraging strong local knowledge and networks, to contribute to community capabilities and resilience.^9^ Their informal delivery mechanisms often ensure resources reach those in need when needed, a potential advantage complementing formal cash transfers or in-kind assistance.

However, since diaspora remittances flow through social networks rather than priority-based allocation systems, this may inadvertently exacerbate inequalities within communities, particularly during crisis. In the aftermath of the tropical storm in Haiti, remittances reached only a small fraction of vulnerable households, primarily those with the strongest migrant connections.^10^ Furthermore, both formal and informal remittance systems depend on financial, telecommunication and transport infrastructure that is often compromised during conflicts or disasters. The Darfur case demonstrates this vulnerability, where remittance flows are disrupted by protracted insecurity, movement restrictions, bank closures and collapse of telecommunication systems.^10^ Finally, remittances have been found to have a limited effect on development in fragile states due to poor policy environments that typically direct these funds towards consumption services rather than sustainable investment.^11^ These nuances highlight the need for an exploration of how best remittances and diaspora financial capital can be leveraged to strengthen the resilience of health systems and communities across all phases: precrisis, in crisis, postcrisis recovery and sustainable development.

## Beyond remittances: innovative financing opportunities

While remittances represent crucial immediate resource flows, diaspora communities contribute financial capital through multiple additional channels, including philanthropy, crowdfunding, private capital investments and resource mobilisation. Diaspora bonds, though untapped in many LMICs, could offer a promising innovative financing mechanism for long-term health commitments. India’s diaspora bonds have raised over $32 billion since inception, partially supporting health infrastructure development.^12^ Similar mechanisms have been proposed as sustainable financing sources for long-term health priorities, including HIV/AIDS programmes in sub-Saharan Africa.^13^

## Diaspora human and social capital: critical resilience capabilities

Diaspora contributions can extend well beyond financial resources. Diasporas mobilise collective resources through philanthropy to community groups and civil society organisations. During the 2014–2016 Ebola outbreak in Sierra Leone, diaspora networks rapidly established the Sierra Leone UK Diaspora Ebola Response Taskforce, which coordinated financial support, technical assistance and health promotion campaigns when traditional aid mechanisms were still mobilising.^14^ Similarly, Syrian diaspora health professional organisations established and resourced health centres that maintained essential services in opposition-held areas.^15^ In conflict-torn Sudan, the Sudanese American Physicians Association supports 13 hospitals and healthcare centres and has delivered more than 57 tons of shipments of essential medications and supplies, effectively preventing the collapse of essential health services in several states.^16^

Complementarily, diaspora human capital is vital for health systems. Diaspora regularly organise medical missions providing free healthcare, run telemedicine services or run capacity-building programmes for local health workers, addressing critical workforce challenges in their countries of origin.^17^ A prominent example of a diaspora-led telemedicine programme is Telekyanmar in Myanmar, initiated following the dual crises of the 2021 military coup and the COVID-19 pandemic, providing critical health services to vulnerable populations in 330 towns in Myanmar.^18^ Diaspora also serve as policy experts or advisors, offering technical expertise to governments, drawing on both intellectual capital and contextual knowledge.

Above all, diaspora communities play a crucial role in building social capital within and between home and host countries. They develop strong social networks that cultivate trust within their communities while also forging institutional collaborations between home and host states. In many instances, they serve as key advocates for their communities of origin, engaging with regional and international actors on issues of human rights, democracy and health equity.

## Diaspora as partners: the path forward

As aid volatility challenges the foundations of health systems in LMICs and fragile settings, diaspora engagement offers a critical yet underutilised opportunity for resilience. To harness this potential fully, three fundamental shifts must occur. First, *recognition*: diaspora must be acknowledged not merely as resource providers but as key health system partners and actors. This recognition necessitates understanding diaspora communities’ unique needs, priorities and collective influence. Second, *integration*: governments should establish dedicated diaspora engagement mechanisms within health ministries, while exploring innovative financing tools like diaspora bonds for sustainable health investments. Although institutionalising diaspora support to health systems can enhance sustainability and integration, context is crucial. Balancing formal structures with informal network structures that have proven effective in crisis response is needed, particularly in fragile settings with complex political environments and contested governance. Finally, *action*: as climate change, conflict and economic instability continue to threaten health systems worldwide, developing evidence-based guidance on operationalising diaspora partnerships for resilient health systems in LMICs and fragile settings represents an urgent priority.

While diaspora communities represent a critical capability for strengthening health systems in LMICs and fragile settings, multiple factors influence the modality, effectiveness and sustainability of these engagements. Known barriers to effective engagement include lack of sustainable follow-through on initiatives, communication gaps between diaspora and origin countries, complex institutional environments, inadequate infrastructure and political instability.^17^ Recent aid cuts have further challenged diaspora civil society organisations, limiting their operational scope and sustainability. This necessitates a nuanced interrogation to develop evidence-based recommendations on operationalising diaspora partnerships for resilient health systems and communities in LMICs and fragile settings.

## Conclusion

In conclusion, the shifting aid landscape requires a reimagining of health financing and partnerships. Recognising and integrating diaspora as key partners is imperative, given their demonstrated financial, human and social capital contributions to resilience of health systems and communities in LMICs and fragile settings. As conventional aid mechanisms face unprecedented volatility, diaspora engagement represents not just a stop-gap measure but a strategic opportunity for strengthening resilient health systems. Fully harnessing this opportunity requires recognition, integration and evidence-based action. This paradigm shift from top-down, aid-dependent programmes to locally set, led, resourced and driven solutions has the potential to strengthen the resilience of health systems and communities and to fundamentally reshape the architecture of global health partnerships.

## Data Availability

All data relevant to the study are included in the article.
